# The Oscillometric Pulse Wave Analysis Is Useful in Evaluating the Arterial Stiffness of Obese Children with Relevant Cardiometabolic Risks

**DOI:** 10.3390/jcm11175078

**Published:** 2022-08-29

**Authors:** Monica Simina Mihuta, Corina Paul, Andreea Borlea, Cristina Mihaela Cepeha, Iulian Puiu Velea, Ioana Mozos, Dana Stoian

**Affiliations:** 1 Department of Doctoral Studies, Victor Babes University of Medicine and Pharmacy, 300041 Timisoara, Romania; 2 Department of Pediatrics, Victor Babes University of Medicine and Pharmacy, 300041 Timisoara, Romania; 3 2nd Department of Internal Medicine, Victor Babeș University of Medicine and Pharmacy, 300041 Timisoara, Romania; 4 Department of Pediatrics, Pius Brinzeu Emergency County Hospital, 300723 Timisoara, Romania; 5 Department of Functional Sciences—Pathophysiology, Center for Translational Research and Systems Medicine, Victor Babeş University of Medicine and Pharmacy, 300173 Timisoara, Romania; 6 Center of Molecular Research in Nephrology and Vascular Disease, Faculty of Medicine, Victor Babes University of Medicine and Pharmacy, 300041 Timisoara, Romania

**Keywords:** arterial stiffness, cardiometabolic risk, childhood obesity, high blood pressure, pulse wave analysis

## Abstract

Early detection of all complications of childhood obesity is imperative in order to minimize effects. Obesity causes vascular disruptions, including early increased arterial stiffness and high blood pressure. This study’s aim is to assess the reliability of pulse wave analysis (PWA) in obese children and how additional risk factors influence the evaluated parameters. We analyzed 55 children aged 6–18 years old by measuring their pulse wave velocity (PWV), augmentation index (AIx), peripheral blood pressure (SBP, DBP), heart rate, central blood pressure (cSBP, cDBP) and central pulse pressure (cPP). We used the oscillometric IEM Mobil-O-Graph and performed a single-point brachial measurement. The subjects were divided into two groups: obese (*n* = 30) and normal-weight (*n* = 25) and were clinically and anamnestically assessed. BMI and waist circumference are significantly correlated to higher values for PWV, SBP, DBP, cSBP, and cDBP. Weight significantly predicts PWV, SBP, DBP and cPP. The risk factors that significantly influence the PWA and BP values are: a cardiometabolically risky pregnancy (higher PWV, AIx, SBP), active and passive smoking (higher PWV, SBP, cSBP, cDBP), sleep deprivation (higher PWV, SBP, cSBP) and sedentariness (higher PWV, AIx, peripheral and central BP). We conclude that obese children with specific additional cardiometabolic risk factors present increased arterial stiffness and higher blood pressure values.

## 1. Introduction

A pandemic that gets worse every year, obesity, is now affecting children more than any generation before [1]. The changes in diet and lifestyle behavior of both children and adults in the last decade has made the fight against obesity a lot harder for physicians all over the world [2,3]. Childhood obesity is a disease that impacts the future wellbeing of more people than any other disease [4]. Obese children become obese adults with various associated pathologies [4]. Out of the many complications that are most likely to occur, the cardiovascular and metabolic complications are the most serious and mutually intricate [5]. Early detection of these complications is therefore imperative to reclaim the fight against obesity and its cardiometabolic adverse effects.

A complication of long-lasting obesity, especially in adults who have had problems with weight excess since their childhood, is early vascular damage. This translates into an early increase in arterial stiffness and a more rapid progression of atherosclerosis [6]. Although the stiffening of the arterial vessels is a natural consequence of aging, metabolic illnesses, arterial hypertension and chronic kidney diseases significantly aggravate this process [7]. Arterial stiffness is one of the major risk factors for cardiovascular disease and an independent risk factor for this type of condition [8].

The gold standard for arterial stiffness assessment is the measurement of pulse wave velocity (PWV) [9]. The PWV is acquired through devices that use tonometry or oscillometry techniques and various measurement points [10,11,12]. It is an easily reproducible technique, great for everyday practice and suitable for children, as well.

The PWV measures the speed at which the pulse wave is transmitted across the length of the arterial tree [13] and assesses the vessel’s elastic properties [4]. The higher the values of the PWV, the stiffer the arteries. A progressively high PWV leads to cardiac remodeling and potentially fatal cardiovascular events [14]. The PWV is estimated by mathematically reconstructing the aortic pulse wave, considering impedance and age. It practically measures the contribution of wave reflection on the arterial pressure waveform [15].

These oscillometric devices also include the measurements of other surrogate markers of vascular health, such as the augmentation index, the peripheral blood pressure, the mean arterial pressure and the heart rate. Some devices even measure the central blood pressure (central systolic blood pressure and central diastolic blood pressure) or the central pulse pressure [15].

The augmentation index (AIx) is also a marker of arterial stiffness and a reliable predictor of cardiovascular events, its higher values being associated to organ damage in adults [16]. It is mainly associated with peripheral arterial resistance [17], which is determined primarily by the elasticity of small arteries and arterioles. In obese and overweight children, multiple studies have shown that the AIx has lower values, probably due to the chronic sympathetic overdrive [18,19,20]. Height is also an important predictor of AIx, smaller heights being associated with higher AIx values [20]. Thus, studies on children should take this into account. The AIx indicates the augmentation component of the aortic systolic blood pressure due to the premature arrival of the reflected wave. The AIx is calculated as the ratio between the central pulse pressure and the reflected pulse pressure, which augments the central BP, called central augmentation pressure [9].

Increased arterial stiffness is associated with increased systolic blood pressure and diastolic blood pressure [21]. Long-lasting obesity in children is associated with a progressive increase in blood pressure [5]. A study by Urbina et.al. on 723 patients aged 10 to 23 (29% of which also presented type 2 DM) showed that in adolescents and young adults with blood pressure values > the 90th percentile, PWV increases progressively [22]. Moreover, mean arterial pressure represents a predictor of PWV, even after correcting for adiposity, metabolic disorders and inflammation [23].

The present study addresses the impact of cardiovascular and metabolic risk factors on the arterial stiffness of obese children with different risk backgrounds. Previous studies have already looked at traditional cardiovascular risk factors such as intrauterine growth restriction, vasculopathies, genetic syndromes associated with vascular disorders, congenital heart disease, diabetes and other endocrine or metabolic diseases, which, to variable degrees have proved to be associated with arterial stiffness progression [24]. We assessed easily identifiable cardiometabolic risk factors by clinical evaluation and targeted anamnesis. Most of these factors are already acknowledged as aggravating factors of cardiovascular and/or metabolic disease in adults but lack exhaustive studies in pediatric populations. For the clinical evaluation, the study focuses on age, sex, puberty development (Tanner stages), body mass index (BMI), waist circumference and blood pressure (BP), factors which are known to be involved in vascular health [25,26,27]. The study also evaluates pregnancy-related risk factors (mother’s health during pregnancy, her weight gain, her smoking), birth weight, postnatal nutrition, family history of cardiometabolic disorders, sedentariness, exposure to cigarette smoke and sleep problems. Our previous work [28] has already shown that a part of these risk factors are linked to increased carotid intima-media thickness (CIMT) and our goal is to provide more data on the links between these factors and vascular health. More studies are needed in order to determine the genetic profile of at-risk patients [29]. It is our aim to provide additional scientific data with regard to childhood obesity and other risk factors’ effects on arterial stiffness progression and ultimately, provide grounds for more efficient preventive measures and for an earlier intervention [30] in such patients.

## 2. Materials and Methods

The study was performed in our Pediatric Endocrinology Department from January 2022 until May 2022 on 55 children. Our work was approved by the Ethics Committee of Scientific Research (CECS) of the University of Medicine and Pharmacy Victor Babes Timisoara (No. 03/19.01.2021) and respects the ethical guidelines of the Helsinki Declaration. Informed consent was acquired from the patient’s legal guardians and verbal agreement was solicited from each child, prior to any examination.

The main objective of this study is to assess the reliability of pulse wave analysis in obese children and how additional cardiometabolic risk factors can influence the analyzed markers. The patients enrolled in the study were split into two groups: obese and normal-weight (N-weight) patients, as controls.

### 2.1. Inclusion Criteria

- Obese group: patients with a BMI score ≥ 95th percentile for age and sex.
- Control group: BMI ranging from the 5th percentile to the 85th.
- Both sexes were included, and ages ranged from 6 to 18.

### 2.2. Exclusion Criteria

Secondary obesity causes: Cushing syndrome, clinical hypothyroidism of any etiology, type 2 DM, polycystic ovarian syndrome, hypothalamic injury/disorders, genetic syndromes such as Prader–Willi syndrome, ghrelin–leptin dysfunction, and use of medication that can induce weight gain (glucocorticosteroids, sulphonylureas, tricyclic antidepressants, antipsychotics) [4].

Other causes for increased arterial stiffness: congenital heart disease, kidney disease, acute inflammatory disorders, oral (dental/gingival) disorders, familial hypercholesterolemia, vasculitis/vasculopathies, type 1 and 2 DM [24].

### 2.3. Clinical Examination, Targeted Anamnesis, Medical History

We performed a clinical examination in all patients, measuring weight, height, BMI, waist circumference and Tanner puberty stage. The blood pressure was acquired through the Mobil-O-Graph device (See Section 2.4).

Similar to our previous work [28], we performed a targeted anamnesis which was focused on the detection of risk factors associated with vascular dysfunction and obesity: postnatal nutrition (breastfed/formula-fed), birth weight (<2500 g/>3500 g/normal weight), pregnancy-associated risk factors (no pathology/>20 kg surplus/gestational diabetes/gestational hypertension/autoimmune thyroiditis), family history (no pathologies/obesity/dyslipidemia/type 2 diabetes/coronary disease/stroke/autoimmune thyroiditis), smoking by the patient (yes/no), physical activity (normal/sedentary) and unhealthy sleep patterns (yes/no). We considered sedentary a subject who performed less than 1 h of physical activity/sport per day. We considered a subject was having an unhealthy sleep pattern when the hours of sleep per night were less than 7 h or when the subject went to bed after midnight during school days.

### 2.4. Arterial Stiffness Assessment Using the Mobil-O-Graph

The evaluation of the arterial stiffness and blood pressure was made using the Mobil-O-Graph^®^ 24 Hour ABPM device (M26101200, IEM^®^ GmbH, Stolberg, Germany). This is a validated, non-invasive device that uses the oscillometric method to perform the pulse wave analysis (PWA) and the blood pressure measurements through a single-point brachial technique. The device has two principal components: the Mobil-O-Graph^®^ PWA and NG with different cuffs and further accessories, and the analysis software for measurement analysis (Hypertension Management Software CS, IEM GmbH, Aachen, Germany). The device can either be used as an outpatient 24-h blood pressure monitoring system and PWA or for a one-time blood pressure and PWA measurement in the physician’s office. We used its latter function for this study. The measurement includes the PWV, the augmentation index (AIx), the systolic blood pressure (SBP), the diastolic blood pressure (DBP), the mean arterial pressure (MAP), the heart rate (HR), the systolic and diastolic central blood pressures (cSBP, cDBP) and the central pulse pressure (cPP). All these parameters were included in our statistical analysis and were called “PWA parameters” in the Results section, for simplification.

This device has a friendly approach to children by making a gentle and fast blood pressure measurement using the Auto Feedback Logic algorithm. Moreover, the software is enhanced with internationally acknowledged pediatric blood pressure limits (American Heart Association).

All measurements were made in the supine position, after at least 10 min of rest in the same position. We advised our patients to abstain from eating, drinking caffeinated drinks or being exposed to cigarette smoke at least 4 h before the measurement. For performing the measurement, we chose appropriate cuff sizes for each patient’s arm circumference (XS: 14–20 cm; S: 20–24 cm; M: 24–32 cm; L: 32–38 cm). The cuff was placed on the patient’s bare upper left arm, with the artery symbol on the cuff positioned on the brachial artery and the pressure tube facing upwards. In order to obtain a correct measurement, the patient must remain completely still and must not speak during the measurement. Dealing with children, it is important to make sure they understand and respect these rules.

This assessment consists of two measurements, separated by a 30-s pause. The first evaluates the blood pressure values and the second performs the PWA and the heart rate, the central blood pressure and pulse pressure measurements. Once the recording is made, the software analyzes the accuracy of the measurement and advises on repeating the measurement if the case requires it. Unless the measurement was considered of poor quality (due to patient’s movements or talking), we made one measurement for each patient. In the cases when the measurement had to be repeated, we allowed the patient to rest for another 5 min before repeating it.

### 2.5. Statistical Analysis

The data were collected and statistically analyzed using Microsoft Excel and MedCalc Statistical Software version 20.111 (MedCalc Software Ltd., Ostend, Belgium). The normality of variable distribution was checked in MedCalc (Shapiro–Wilks test): means, Student’s T test and Pearson’s correlations were used for normally distributed variables and medians, the Mann–Whitney test and Spearman’s correlations were used for non-normally-distributed variables.

Subjects were divided into two groups—obese and controls (normal-weight)—and multiple subgroups based on age, sex, Tanner stage, risk factors. These categories were analyzed focusing on: PWV (m/s), AIx %, SBP (mmHg), DBP (mmHg), MAP (mmHg), HR (b/min), cSBP (mmH), cDBP (mmHg), cPP (mmHg).

Statistical significance was considered *p* < 0.05. For cases of multiple analysis on the same data (2 by 2 tests on three groups), we performed a one-way ANOVA post hoc (single factor) test in Microsoft Excel and adjusted the *p*-values according to the Bonferroni-corrected α in order to keep the significance threshold below 0.05. Multivariable regression analysis was performed in MedCalc with the stepwise method, with each above-mentioned PWA parameter taking its turn as the dependent variable.

## 3. Results

### 3.1. Descriptive Analysis of the Data

The study was performed on 55 children of both sexes, ages 6 to 18. They were divided into two group studies, based on their BMI: obese (13 girls and 17 boys), with a median BMI of 26.5 kg/m^2^ and normal weight (13 girls and 12 boys), with a median BMI of 18.6 kg/m^2^. All variables were tested for distribution normality (Table 1 and Table 2).

### 3.2. Pulse Wave Analysis with Regard to BMI

**Figure 1:**
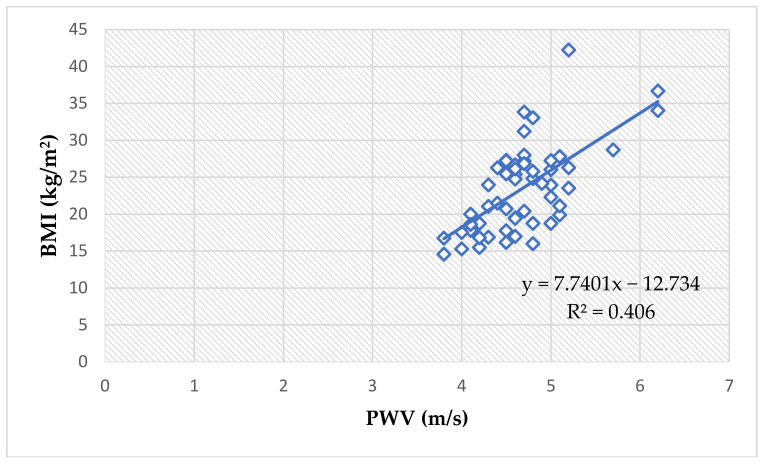
The correlation between PWV values and BMI values in all children (Spearman’s ρ = 0.58, *p* < 0.0001).

Most PWA parameters showed very significant correlations to BMI (Table 3, Figure 1), thus validating the study’s aim to assess these parameters in pediatric patients. Pearson’s correlation was used for normally distributed variables and Spearman’s correlation for the non-normally distributed ones; each parameter was tested for normality of distribution before we applied the statistical tests.

### 3.3. Pulse Wave Analysis with Regard to Sex

The analysis included 13 obese girls (43.3%) and 17 obese boys (56.6%) and 13 normal-weight girls (52%) and 12 normal-weight boys (48%). Table 4 shows mean and median values for each subgroup and *p*-values for the comparisons between analyzed data.

We detected significant differences in PWA parameters when comparing same-sex subjects from obese vs. control groups (Table 5).

We mention that the results presented in Table 4 and Table 5 were obtained by performing *t*-Student tests and Mann–Whitney tests, after checking each parameter’s normality of distribution.

### 3.4. Pulse Wave Analysis with Regard to Age

Each group of study was divided into three subgroups according to age: <12 years old, 12–15 years old and ≥16 years old. The one-way ANOVA test detected no differences across the three subgroups of obese subjects concerning PWV (*p* = 0.21), AIx (*p* = 0.99), SBP (*p* = 0.26), DBP (*p* = 0.05), MAP (*p* = 0.09), HR (*p* = 0.51), cSBP (*p* = 0.22), cDBP *(p* = 0.06), cPP (*p* = 0.86). Although the ANOVA test detected a significant difference across the three normal-weight subgroups for PWV (*p* = 0.04) and SBP *(p* = 0.04), the Bonferroni-corrected *p*-values did not meet statistical significance levels. No differences were detected in normal-weight subgroups for AIx (*p* = 0.79), DBP (*p* = 0.42), MAP (*p* = 0.06), HR (*p* = 0.76), cSBP *(p* = 0.25), cDBP (*p* = 0.09), cPP (*p* = 0.22).

As depicted in the Table 6, most PWA parameters analyzed in the obese group presented higher values. However, when comparisons across the three subgroups divided by age were made, no significant differences were detected after the Bonferroni corrections.

Comparisons between obese and normal-weight children of the same ages were made by performing *t*-Student tests and Mann–Whitney tests after checking each parameter’s normality of distribution and showed significantly higher values for PWV (*p* = 0.03), cSBP (*p* = 0.005) and cPP (*p* = 0.02) in obese children <12 years old (Table 7), and for PWV (*p* = 0.007), SBP *(p* = 0.03), HR (*p* = 0.03), cSBP (*p* = 0.003) and cDBP (*p* = 0.002) in obese children aged 12 to 15 years old (Table 8).

The analysis of the ≥16 years old subjects is statistically unreliable, as the obese group counts six subjects and the normal-weight group just three subjects. Nevertheless, no significant differences were detected between obese and controls ≥ 16 years old.

### 3.5. Pulse Wave Analysis with Regard to Tanner Puberty Development Stages

Each group of study was divided into three subgroups according to Tanner stages for puberty development: one group included Tanner stage I, one group included Tanner stages II and III, one group included Tanner stages IV and V. The one-way ANOVA test revealed no significant differences with regard to PWA parameters across the three subgroups, except for the cDBP, which was significantly higher in Tanner stage IV and V children (*p* = 0.04, Bonferroni-corrected). Nevertheless, values of the analyzed parameters are higher in the subjects with Tanner IV and V development in all cases, except HR, as can be seen in Table 9.

The analysis of PWA parameters of the three subgroups divided by Tanner stages showed significantly higher values for cSBP (*p* = 0.006), cDBP (*p* = 0.02) in Tanner I obese children compared to their controls (Table 10). Moreover, cSBP was also higher in the Tanner II and III obese subjects (*p* = 0.02, Table 11), and in the Tanner IV and V obese subjects (*p* = 0.02), along with cDBP (*p* = 0.002, Table 12). We mention that the results presented in Table 10, Table 11 and Table 12 were obtained by performing *t*-Student tests and Mann–Whitney tests, after checking each parameter’s normality of distribution.

### 3.6. PWA and the Waist Circumference

The mean waist circumference for obese children was 97.6 cm (Table 1). We detected positive correlations between the waist circumference of obese children and their PWV, SBP, DBP, MAP, cSBP and cDBP (Table 13). The mean waist circumference for normal-weight children was 63.7 cm (Table 2). We detected positive correlations between the waist circumference of obese children and their PWV, SBP, MAP and cSBP (Table 13).

**Figure 2:**
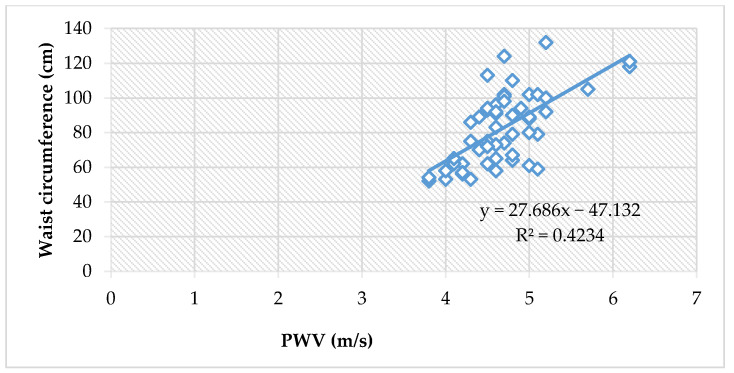
The correlation between PWV and waist circumference, in all children (Spearman’s ρ = 0.62, *p* < 0.0001).

Moreover, the waist circumference showed an even more significant positive correlation to PWV values when assessing all children (ρ = 0.62, *p <* 0.0001), Figure 2.

**Figure 3:**
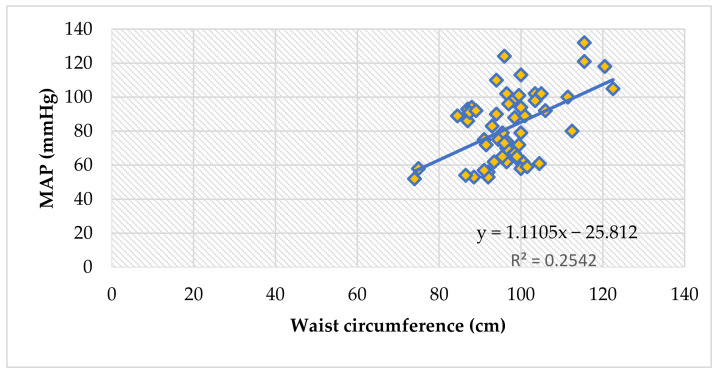
The correlation between mean arterial pressure and waist circumference, in all children (Spearman’s ρ = 0.42, *p* = 0.001).

The waist circumference also reliably correlates to peripheral blood pressure values (systolic and diastolic), and thus, with the mean arterial pressure values, in all children (ρ = 0.42, *p* = 0.001), Figure 3.

### 3.7. The Assessment of Risk Factors

The risk factors assessment was made by comparing subgroups that have been exposed to risks vs. subgroups that have not.

#### 3.7.1. PWA and Risk Factors in the Obese Group

The obese subjects presented significantly higher PWV (*p* = 0.02), AIx (*p* = 0.009) and SBP (*p* = 0.01) values in children coming from mothers with cardiometabolic risk during pregnancy. No differences were detected for birth weight, post-natal nutrition and family history of cardiometabolic risk. Smoking increases PWV (*p* = 0.01) and SBP (*p* = 0.15) values significantly. We did not detect any differences between smoking and passive smoking in the obese group. Children who were passively smoking did present lower values for PWA parameters than smokers and higher than non-smokers, but the analysis did not detect statistical significance. The subjects who declared problems with sleeping presented significantly higher values for PWV (*p* = 0.004), SBP (*p* = 0.008), MAP (*p* = 0.04) and cSBP (*p* = 0.01). See Table 14.

**Figure 4:**
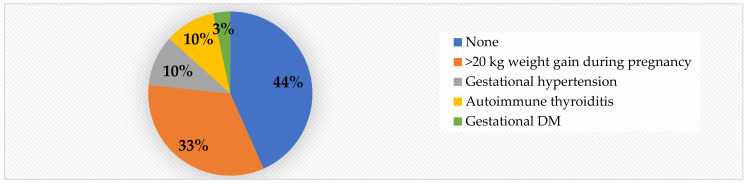
Mother’s health during pregnancy in obese children.

With regard to maternal risk factors, we noted that 56.6% of the subjects were born to unhealthy mothers. Out of the total cases, 33% gained >20 kg during pregnancy, making this the most prevalent risk factor we have encountered. Gestational hypertension and autoimmune thyroiditis were detected in 10% of the cases each, while gestational DM was present in only 3% of the cases (Figure 4).

**Figure 5:**
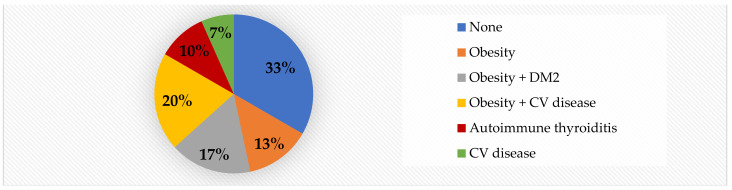
Family history of risk factors in obese children.

Although we did not detect significant differences with regard to family history of cardiometabolic risk, it is worth noting that obesity with/without other associated diseases such as type 2 DM and cardiovascular diseases (coronary disease, arterial hypertension, stroke) was detected in 50% of cases, while 67% of all obese children had a positive family history for cardiometabolic risk (Figure 5).

#### 3.7.2. PWA and Risk Factors in the Normal-Weight Group

In the control group, we detected significantly higher PWA parameters (all, except cPP) in children exposed to cigarette smoke. All the parameters of the PWA were significantly higher in the sedentary normal-weight children. The normal-weight subjects with sleep problems presented significantly higher values for PWV (*p* = 0.04), SBP (*p* = 0.04), DBP MAP (*p* = 0.01) and cPP (*p* = 0.02). Although overall values for PWA parameters were higher in children with other risk factors, statistical significance was not met. See Table 15.

**Figure 6:**
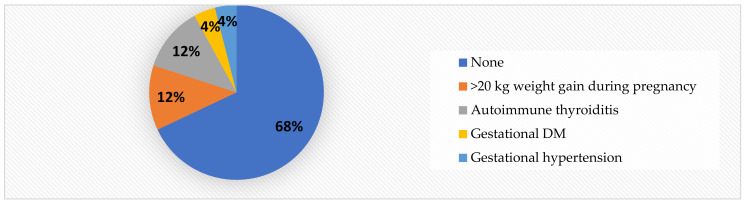
Mother’s health during pregnancy in normal-weight children.

It is worth mentioning that, in contrast to the obese children (See Figure 4), 68% of the normal-weight patients were born to healthy mothers. Only 12% of the mothers had a >20 kg weight gain during pregnancy (Figure 6), compared to 33% in the obese group.

### 3.8. The Multilinear Regression Model

A multivariate linear regression analysis (stepwise method) on the entire lot (*n* = 55) was performed in order to identify independent predictors for PWA parameters. The independent variables were weight, height, BMI and waist circumference, and the dependent variables were each of the PWA parameters, in turn.

The weight represents a significant predictor of PWV (*p <* 0.0001, R^2^ = 0.54, t = 7.93), SBP (*p* < 0.0001, R^2^ = 0.44, t = 6.47), cSBP (*p <* 0.0001, R^2^ = 0.47, t = 6.97), cDBP (*p <* 0.0001, R^2^ = 0.41, t = 6.07) and cPP (*p <* 0.01, R^2^ = 0.11, t = 2.61).

The height is a significant predictor of AI (*p* = 0.01, R^2^ = 0.1, t = 2.4), DBT (*p* = 0.0009, R^2^ = 0.08, t = 3.5) and MAP (*p* = 0.003, R^2^ = 0.15, t = 3.11).

The BMI predicts HR significantly (*p* = 0.0323, R^2^ = 0.08359, t = 2.199).

The waist circumference, although significantly correlated to PWV, SBP, DBP, MAP, cSBP and cDBP (Table 13), is not a significant predictor of any of these parameters.

## 4. Discussion

The purpose of this study was to evaluate the potential usefulness of the pulse wave analysis in obese children with additional cardiometabolic risk. Our aim was to show that pulse wave analysis can be considered a reliable evaluation method in obese children, as previously described and demonstrated in obese adults [8], as it gives the physician additional information about the patient’s vascular health. The analysis was focused on the influence of genetic and epigenetic risk factors on PWV, AIx, SBP, DBP, MAP, HR, cSBP, cDBP, and cPP.

We chose the IEM Mobil-O-Graph device to perform our study, because it is a study-validated device which includes the measurement of central blood pressure. It is also a user-friendly, time-saving device and it is recommended by the manufacturer for pediatric use, in children older than 3 years old. The device has been validated in adults against the well-established SphygmoCor applanation tonometry device, combining the measurement of brachial BP, pulse wave analysis and central BP in one measurement [31,32,33]. Although the oscillometric noninvasive methods of acquiring this data are not completely validated in children, there are multiple studies that affirm that these devices’ accuracy is comparable to mercury sphygmomanometry [34,35,36]. The main issue seems to be the accuracy of the systolic peripheral BP and the central systolic BP, which, according to Mynard et al., could be overestimated [37].

Atherosclerosis is a process that starts in early childhood and progresses more or less rapidly, depending on genetic and environmental factors [38]. In patients under 18 years old that present cardiovascular and/or metabolic risk of any etiology, and even in young adults, we expect to objectify a subclinical atherosclerosis that manifests in higher arterial stiffness [7,23] and a higher intima-media thickness [28]. How rapidly the subclinical atherosclerosis turns into a clinical vascular dysfunction depends on the individual genetic substrate and the power of the epigenetic factors to which the individual is exposed. The PWA and the central blood pressure monitoring of patients at risk are tools that can be useful in assessing the severity of vascular dysfunction over time, and thus, to ensure proper and timely medical intervention in order to reduce the severity of outcome [39].

The analysis was made on two groups: obese children and normal-weight children. Each clinical and anamnestic parameter we followed was in relation to the pulse wave analysis we performed with the Mobil-O-Graph device.

The study’s main premise is that excess adipose tissue increases arterial stiffness, hence the analysis began with identifying correlations between weight, height and BMI and PWA parameters. As depicted in Table 3, the PWV, SBP, cSBP and cDBP are all extremely significantly correlated to the three clinical parameters, with p-values of 0.0001 or smaller. DBP and MAP are showing more moderate positive correlations, while cPPis correlated only to weight and BMI. Heart rate did not correlate to any of the three parameters.

Other studies have also shown that obesity is an independent predictor of PWV and peripheral blood pressure values in children [19,20,22]. Weight is a significant predictor of PWV (*p* < 0.0001), SBP (*p* < 0.0001), cSBP (*p* < 0.0001), cDBP (*p* < 0.0001) and cPP *(p* = 0.0117). However, central BP values are not well-validated with regard to normal ranges in children, and questions are raised with regard to the possibility of tonometry and oscillometry underestimating central BP [40,41], thus making the use of these methods of measurement questionable with regard to their clinical use. Our results, nevertheless, show strong positive correlations between central BP values and BMI, which suggests that even if the values are underestimated, adipose tissue still has an impact on central BP. More studies are needed for entirely validating the data. It is worth mentioning that height has a well-established inversed effect on these vascular parameters in adults [42,43,44], but not in children who have not yet completed their statural growth. Due to the fact that weight itself showed significant correlations to the same parameters, we can affirm that BMI does indeed show reliable correlations to our measurements. Our analysis showed that height is a significant predictor of AI (*p* = 0.0161), DBP (*p* = 0.0009) and MAP (*p* = 0.003).

Sex is a very debatable factor in the progression of arterial stiffness. A study on gender differences in large arteries stiffness of pre- and post-pubertal subjects has suggested that arterial stiffness varies between genders: pre-pubertal girls showed higher values for PWV and pulse pressure than pre-pubertal boys, but post-pubertal males showed significantly stiffer vessels than age-matched females [45]. These differences could be accounted for by males being taller, heavier and having a larger cardiac output [45], but sexual hormones in both sexes are also very important factors, as sex-hormone receptors for androgens, estrogen and progesterone are expressed in the blood vessels, female hormones having a well-established cardiovascular protective role [46,47]. Postmenopausal women present disproportionately stiffer vessels than same-age men [48,49] and double the cardiovascular risk [45]. It is worth mentioning that while in the 1980s, and even 1990s, testosterone was known as a promoter of cardiovascular disease [50], recent studies have deconstructed this theory, as it was shown that testosterone plays a positive role in arterial reactivity [51] and compliance [52] and is a negative predictor of arterial stiffness in men even after adjusting for risk factors such as age, pulse pressure, BMI and total cholesterol [53]. Our study found higher values for AI (*p* = 0.015) and HR *(p* = 0.005) for obese girls, in comparison to obese boys (Table 4). In comparing each sex (obese vs. normal weight), we found that excess weight significantly increases the values of PWV, SBP, cSBP and cDBP (Table 5).

The arterial stiffness is influenced by aging but the mechanisms behind it are only partially known. The alterations in collagen and the decrease in elastin seem to be the main culprits [54]. In children and young adults, age could be less involved in the process of vascular damage than individual pathologies such as obesity and exposure to cardiometabolic risks. An Argentinian study on 780 cardiometabolically healthy people, aged 10 to 98, showed that the PWV is strongly correlated to age, but that in younger subjects the dispersion is much lower [55]. Meanwhile, with regard to blood pressure, a Chinese study on 1066 women and 978 men addressing the impact of age on peripheral and central BP showed that for young subjects, age does have an influence on cSBP [56]. Our study detected no differences for any of the PWA parameters across the three subgroups (<12 years old, 12–15 years old and ≥16 years old), for either obese or normal-weight children (Table 6). Comparisons between obese and normal-weight children of the same ages showed significantly higher values for PWV, cSBP and cPP in obese children <12 years old (Table 7), and for PWV, SBP, HR, cSBP and cDBP in obese children aged 12 to 15 years old. We could not perform an analysis for children 16 and older because the lots were too small.

Since age is not a reliable predictor of risk in children, we also analyzed the data from the perspective of puberty development (Tanner stages). A study on obese adolescents showed that Tanner development stages are not correlated to PWV or AI [57]. The expected differences should appear if the theory that androgens have a damaging effect on the arterial wall were true. As mentioned before, studies have shown that testosterone plays a positive role, at least in boys [53]. However, obese adolescent girls do generally present higher levels of androgens and this fact could interfere with cardiovascular health [58]. In our study, the ANOVA test did not detect any significant differences across the three subgroups (Tanner I, Tanner II and III, Tanner IV and V) for each PWA parameter. Overall values of all parameters except heart rate were indeed higher in Tanner IV and V children, however, after the Bonferroni correction, we could not present any significant differences. When we compared same puberty stage children (obese vs. normal-weight), we did find significantly higher values for cSBP *(p* = 0.006) and cDBP *(p* = 0.021) in Tanner I obese children compared to their controls (Table 10), a higher value for cSBP in the Tanner II and III obese subjects (*p* = 0.026, Table 11) and higher values for cSBP (*p* = 0.028) and cDBP *(p* = 0.0029, Table 12) in Tanner stage IV and V children.

Waist circumference is a clinical tool that very accurately estimates visceral adipose tissue in the abdomen. Not only is it a key component of the metabolic syndrome [59], but it is reliably linked to insulin-resistance and cardiovascular events [60]. We detected positive correlations between the waist circumference of obese children and their PWV, SBP, DBP, MAP, cSBP and cDBP (Table 13, Figure 2 and Figure 3).

With regard to risk factors, the analysis was focused on anamnestically identifiable risks. We detected significantly higher PWV in obese children born to mothers who have been cardiometabolically unhealthy during pregnancy (*p* = 0.02), in obese adolescents who were smokers *(p* = 0.013) and in obese children with sleeping issues (*p* = 0.004). Additionally, in the obese group, we found significantly higher values for AIx (*p* = 0.009) and for SBP (*p* = 0.01) in patients born to unhealthy mothers, and for SBP in smokers (*p* = 0.015). Moreover, children with sleep problems also presented higher values for SBP (*p* = 0.008), MAP (*p* = 0.04), and cSBP (*p* = 0.017), see Table 14. Over a half (56%) of the obese patients were born from cardiometabolically unhealthy pregnancies, while only 32% of the normal-weight children did the same. The most prevalent pregnancy risk factor was gaining >20 kg during pregnancy (33% in the obese group, compared to 12% in the control group). Gestational hypertension and autoimmune thyroiditis were detected in 10% of the cases each, while gestational DM was present in only 3% of the cases (Figure 2).

A mother’s health during pregnancy is related to the development of the foetus and the future of the child [61]. Maternal obesity, the most prevalent pregnancy-associated risk in our study, causes placental insufficiency which leads to intrauterine growth delay [62] and cardiovascular alterations that can become permanent predispositions for high blood pressure, arterial stiffness and atherosclerosis [63].

Smoking affects the integrity of the vascular wall, promotes early atherosclerosis and accelerates the arterial stiffness [64]. It is worth noting that, in the obese group, although significance was not met, passive smokers presented higher median/mean values for PWV and peripheral and central BP compared to children not exposed to smoke. No differences in these parameters were detected in comparison to smokers. In the control group, children exposed to smoking presented significantly higher values for PWV (*p* = 0.02), AIx (*p* = 0.009), SBP (*p* = 0.03), DBP (*p* = 0.01), MAP (*p* = 0.02), cSBP (*p* = 0.03) and cDBP (*p* = 0.02). This is yet another argument that passive smoking is as bad as smoking for vascular health.

Sleep deprivation in children is a very complex matter, that is usually associated with intellectual, behavioral and emotional problems [65,66]. The National Heart, Lung and Blood Institute recommends 10 h of sleep a day for school-aged children [67], but epidemiological studies show that children sleep much less than that: 31% of American children aged 6 to 11 years sleep less than 9 h per night [68], 33% of European teenagers sleep less than 8 h [69], and adults sleep 6 h or less [70]. Multiple studies on adults show that sleep dysfunctions have detrimental effects on the cardiovascular and metabolic systems, apart from the neuro-psychological and social effects. Acute sleep deprivation is associated with increased PWV and AI, both markers of arterial stiffness, in young adults [71]. A study on young to middle-aged adults with obesity showed that short sleep duration is associated with higher PWV values [72], while a study on older subjects showed that poor sleep quality is associated with higher PWV as well [73]. Blood pressure is known to be higher or harder to control in all types of sleep disorders, from psychiatric pathologies to sleep apnea, behavioral short sleep duration or shift work [74,75]. Multiple studies have linked sleep deprivation with high blood pressure in children as well [76], thus it can accurately be considered a cardiovascular risk factor. Our study confirms both the higher arterial stiffness markers and the higher BP values in children with sleep problems. In addition to the significant results detected in the obese group, mentioned above, the normal-weight subjects with sleep issues presented significantly higher values for PWV (*p* = 0.04), SBP *(p* = 0.04), DBP (*p* = 0.01), MAP (*p* = 0.01) and cPP *(p* = 0.02), as well. These results show that sleep, often a neglected part of our patient management, is a very relevant factor in the fight against the progression of cardiometabolic diseases. Most of our subjects reported elective insufficient sleep. None reported physical problems such as sleep apnea, chronic diseases associated with pain or poor-controlled asthma. The vast majority presented unhealthy sleep patterns due to activities related to virtual communication, social media, video-games, watching TV and other related activities. Five adolescents reported stress-related sleep difficulties. Hence, since both obese and normal-weight children presented results that can be associated with cardio-vascular risk, we suggest that sleep assessment should have a more impactful role in our everyday medical activity.

Our study did not detect any differences for subjects with small or large birth weight compared to normal birth-weight, in either obese subjects or controls. However, multiple studies show that in low-birth-weight children, the fetal growth restriction is an important factor for the child’s cardiovascular risk in adulthood [77]. On the other hand, large birth weight is associated with a predisposition to obesity and is indirectly linked to cardiovascular risk [78].

Breastfeeding has a well-established cardio-protective role, provided it is not prolonged [79]. Its connection to vascular health includes: a decrease in atherosclerosis progression and a preservation of normal arterial elasticity [80]. Our present study did not detect any differences with regard to artificial vs. natural postnatal nutrition, but our previous work did connect artificial feeding with higher intima-media thickness in overweight children [28].

We did not detect significant differences with regard to family history of cardiometabolic risk. However, 67% of all obese children had a positive family history for cardiometabolic risk (50% obesity +/− other associated diseases such as type 2 DM and cardiovascular diseases—coronary disease, arterial hypertension, stroke—7% cardiovascular disease in the absence of obesity, and 10% autoimmune thyroiditis), see Figure 5. Despite our results, studies show very clearly that family history of obesity influences the onset and the severity of childhood obesity [81] and the severity of obesity (expressed through BMI) is directly linked to increased arterial stiffness in children [82].

The lack of everyday physical activity in children is detrimental to both the metabolic and the cardiovascular systems [83]. In the obese group, we did not detect any differences in PWA parameters when comparing sedentary subjects to subjects having normal physical habits. However, we did detect very significant differences in normal-weight children: normal-weight sedentary subjects presented significantly higher values for all PWV parameters (Table 15). This goes to show that in the absence of the metabolic imbalance caused by obesity and all its complications, the lack of daily physical activity has a strong impact on vascular health.

A particularity worth mentioning is that in this study, the heart rate did not present an evident, clear pattern in relation to the analyzed parameters. Some obese subgroups presented higher HR values, such as obese girls vs. obese boys (*p* = 0.005, Table 4), obese girls vs. normal-weight girls (*p* = 0.004, Table 5) or obese 12–15-year-olds vs. their normal-weight counterparts (*p* = 0.03, Table 8). Certainly, apart from the obese subjects, younger children presented higher values for HR throughout the subgroups, but without statistical significances. This finding agrees with previous studies which have shown that HR has an inversed relationship with age [84,85]. This is explained by the fact that infants and young children have increased sympathetic and parasympathetic activity, whereas adolescents present a decrease of sympathetic activity and a slight decrease of parasympathetic one [86]. Moreover, although weight gain is associated with an increase of sympathetic activity [86], long-lasting obesity in children leads to a significant decrease [86,87]. Additionally, in young children we cannot measure how much of the increase in HR values is due to the sympathovagal imbalance and how much is due to the physician’s office-related nerves. These particularities may explain why our study has not found correlations between HR values and clinical parameters (Table 3 and Table 13) or with risk factors (Table 14). It may be a limitation of our study that we have not investigated the duration of the obesity.

Other important limitations of this study are mainly linked to the small sizes of the study groups, which in some cases interfered with the statistical analysis (for normal-weight subjects who presented <2500 g birth weight or who were smokers). We believe that a further analysis of birth weight, postnatal nutrition and family history on a larger number of subjects might lead to a more accurate conclusion regarding risk. Additionally, we acknowledge that adding the measurement of the flow-mediated dilation (FMD) to our research would have improved the assessment of the cardiovascular risk. FMD is a non-invasive, easily reproducible, ultrasonography measurement, considered a gold-standard for the detection of early vascular dysfunction [88]. Previous studies have shown that weight gain in children is associated with a lower FMD [89], before any ultrasonography-detectable atherosclerotic changes occur in the arterial walls [90].

A longitudinal study regarding the PWA and central BP dynamics over time and the influence of losing weight, maintaining or gaining more weight on these parameters, would be valuable.

## 5. Conclusions

Arterial stiffness and blood pressure values are aggravated by weight excess in children. The higher the BMI and the waist circumference, the higher the PWV, peripheral and central BP values. Weight is a significant predictor of PWV, peripheral BP, central BP and central pulse pressure.

Obese children born from pregnancies with high cardiometabolic risk present significantly higher PWV, AIx and SBP. The most-prevalent risk factor related to pregnancy is >20 kg weight gain.

Smoking in obese adolescents is linked to higher PWV and SBP values. Normal-weight children exposed to passive smoking present significantly higher values for PWV, peripheral and central BP.

Sleep deprivation is associated to higher PWV, SBP, MAP and cSBP values in both obese and normal-weight children.

Sedentariness is associated with significantly higher PWA parameters.

The AIx is inconsistently affected by the evaluated clinical parameters and risk factors, and we conclude that more research is needed for a better understanding of its dynamic.

## Figures and Tables

**Table 1:** Normality and descriptive analysis of variables in the obese group (*n* = 30).

Shapiro–Wilks Test for Normality of Distribution	Accepted Normality *p*-Value	Rejected Normality *p*-Value	Mean	95% CI	Median	95% CI	SD
BMI (kg/m^2^)	-	0.0003	-	-	26.5	25.7–27.3	4.41
Weight (kg)	-	0.03	-	-	63	59.2–73.6	21.5
Height (m)	0.22	-	1.55	1.5–1.64	-	-	0.15
Waist circumference (cm)	0.38	-	97.61	92.42–102.51	-	-	13.9
Weight at birth (g)	0.11	-	3058	2818–3297	-	-	642.52
PWA (m/s)	-	0.0003	-	-	4.7	4.61–4.92	0.47
AIx %	0.65	-	24.82	20.61–28.92	-		11.12
SBP (mmHg)	-	0.004	-	-	118	114.1–125	11.88
DBP (mmHg)	0.85	-	76.49	72.51–80.43	-	-	10.56
MAP (mmHg)	0.07	-	99.31	95.6–103	-	-	9.96
cSBP (mmHg)	-	0.006	-	-	113	107–117	11.36
cDBP (mmHg)	0.63	-	78.12	74.3–81.89	-	-	10.12
cPP (mmHg)	0.35	-	37.11	33.21–41.02	-	-	10.47
HR (b/min)	0.5	-	89.31	85.2–93.52	-	-	11.08

**Table 2:** Normality and descriptive analysis of variables in the normal-weight group (*n* = 25).

Shapiro–Wilks Test for Normality of Distribution	Accepted Normality *p*-Value	Rejected Normality *p*-Value	Mean	95% CI	Median	95% CI	SD
BMI (kg/m^2^)	-	<0.0001	-	-	18.6	16.82–19.52	4.22
Weight (kg)	0.22	-	41.32	36–46.7	-	-	12.71
Height (m)	-	0.02	-	-	1.52	1.41–1.52	0.18
Waist circumference (cm)	0.23	-	63.71	60.42–67.11	-	-	8.21
Weight at birth (g)	0.94	-	3322	3037.5–3606.4	-	-	689
PWA (m/s)	0.33		4.38	4.2–4.6	-	-	0.43
AIx %	0.51	-	21.82	17.7–25.9	-	-	9.79
SBP (mmHg)	0.05	-	113.63	109.9–117.3	-	-	8.91
DBP (mmHg)	-	0.002	-	-	78	75–81	8.16
MAP (mmHg)	-	0.02	-	-	96.5	92.21–98.89	8.1
cSBP (mmHg)	0.29	-	100.31	96–104.6	-	-	10.42
cDBP (mmHg)	0.56	-	68.32	64.8–71.7	-	-	8.38
cPP (mmHg)		0.02	-	-	31	29.12–33.81	5.73
HR (b/min)	0.61	-	83.74	81–86.44	-	-	6.51

**Table 3:** Correlations between each PWA parameter and BMI, weight and height, respectively, in all children (*n* = 55).

	PWV m/s	Aix %	SBP mmHg	DBP mmHg	MAP mmHg	HR b/min	cSBP mmHg	cDBP mmHg	cPP mmHg
BMI (kg/m^2^)	**0.58**	0.21	**0.49**	**0.26**	**0.31**	0.24	**0.64**	**0.56**	**0.28**
*p*-value	**<0.0001**	0.11	**0.0001**	**0.04**	**0.02**	0.07	**<0.0001**	**<0.0001**	**0.03**
Weight (kg)	**0.68**	**0.29**	**0.61**	**0.36**	**0.35**	0.22	**0.67**	**0.59**	**0.30**
*p*-value	**<0.0001**	**0.03**	**<0.0001**	**0.006**	**0.008**	0.1	**<0.0001**	**<0.0001**	**0.02**
Height (m)	**0.58**	**0.25**	**0.57**	**0.43**	**0.3**	0.03	**0.5**	**0.51**	0.2
*p*-value	**<0.0001**	**0.06**	**<0.0001**	**0.001**	**0.01**	0.79	**0.0001**	**<0.0001**	0.13

The numbers in bold represent statistically significant results.

**Table 4:** PWA parameters by sex.

Group	Sex	PWV m/s	AIx %	SBP mmHg	DBP mmHg	MAP mmHg	HR b/min	cSBP mmHg	cDBP mmHg	cPP mmHg
Obese	Boys	4.75	19.31	120.31	75.93	98.12	85.06	113.68	76.68	41.5
	Girls	4.78	30.3	117	75	97	95.53	112.84	79.07	33.93
*p*-value		0.54	**0.01**	0.89	0.98	0.9	**0.005**	0.43	0.66	0.6
N-weight	Boys	4.45	22.41	114.33	76.83	95.58	83.91	100.58	68	32
	Girls	4.4	21.23	115	76	96.5	83.53	100.15	68.53	31.07
*p*-value		0.6	0.7	0.7	0.49	0.74	0.88	0.27	0.87	0.33

The number in bold represents statistically significant results.

**Table 5:** PWA comparison for the same sexes.

	PWV m/s	AIx %	SBP mmHg	DBP mmHg	MAP mmHg	HR b/min	cSBP mmHg	cDBP mmHg	cPP mmHg
Obese girls	4.78	30.3	117	75	97	95.53	112.84	79.07	33.93
N-weight girls	4.4	21.23	115	76	96.5	83.53	100.15	68.53	31.07
*p*-value	**0.016**	**0.02**	0.09	0.93	0.39	**0.004**	**0.006**	**0.01**	0.39
Obese boys	4.75	19.31	120.31	75.93	98.12	85.06	113.68	76.68	41.5
N-weight boys	4.45	22.41	114.33	76.83	95.58	83.91	100.58	68	32
*p*-value	**0.03**	0.43	0.1	0.81	0.46	0.69	**0.001**	**0.02**	0.09

The numbers in bold represent statistically significant results.

**Table 6:** Mean and Medians of PWA parameters for the obese subjects, by age.

	*n*	PWV m/s	AIx %	SBP mmHg	DBP mmHg	MAP mmHg	HR b/min	cSBP mmHg	cDBP mmHg	cPP mmHg
<12 years old	11	4.5	25	118.91	70.91	94.91	91.81	111.63	73.09	38.36
12–15 years old	13	4.8	24.53	119	78.38	100.11	89.15	117	79.53	36
≥16 years old	6	4.9	25.16	123.5	82.66	102.25	85.16	117.5	84.33	37.16

**Table 7:** PWA parameters comparison in subjects <12 years old.

		*n*	PWV m/s	AIx %	SBP mmHg	DBP mmHg	MAP mmHg	HR b/min	cSBP mmHg	cDBP mmHg	cPP mmHg
<12 years	Obese	11	4.5	25	118.91	70.91	94.91	91.81	111.63	73.09	38.36
	N-weight	12	4.2	21	109.41	73.5	91.45	83.75	96.5	66.5	30.5
	*p*-value		**0.03**	0.38	0.06	0.55	0.41	0.052	**0.005**	0.12	**0.02**

The numbers in bold represent statistically significant results.

**Table 8:** PWA parameters comparison in subjects aged 12–15 years old.

		*n*	PWV m/s	Aix %	SBP mmHg	DBP mmHg	MAP mmHg	HR b/min	cSBP mmHg	cDBP mmHg	cPP mmHg
12–15 years	Obese	13	4.8	24.53	121.8	78.38	100.11	89.15	113.76	79.53	36
	N-weight	10	4.5	20.7	115.8	77.4	96.6	82.3	102.8	67.9	33.6
	*p*-value		**0.007**	0.42	**0.03**	0.77	0.2	**0.03**	**0.003**	**0.002**	0.48

The numbers in bold represent statistically significant results.

**Table 9:** Mean and medians of PWA parameters for the obese patients, by Tanner stages.

Tanner Stage	PWV m/s	AIx %	SBP mmHg	DBP mmHg	MAP mmHg	HR b/min	cSBP mmHg	cDBP mmHg	cPP mmHg
I	4.5	22.31	110.02	74.22	95.7	91.42	110	75.81	34.54
II, III	4.8	23.52	121.73	73	97.39	88.71	114.33	74.62	39.21
IV, V	4.85	28.33	126.19	82.5	104.3	88.74	118	84.32	36.14

**Table 10:** PWA parameters comparison in Tanner Stage I subjects.

Tanner I Subjects	*n*	PWV m/s	AIx %	SBP mmHg	DBP mmHg	MAP mmHg	HR b/min	cSBP mmHg	cDBP mmHg	cPP mmHg
Obese	7	4.5	22.31	110.02	74.22	95.7	91.42	110	75.81	34.54
Normal weight	9	4.1	17.4	109	72	89.2	83.7	92	63.3	29
*p*-value		**0.01**	0.32	0.18	0.67	0.25	0.17	**0.006**	**0.02**	0.13

The numbers in bold represent statistically significant results.

**Table 11:** PWA parameters comparison in Tanner Stage II and III subjects.

Tanner II, III Subjects	*n*	PWV m/s	Aix %	SBP mmHg	DBP mmHg	MAP mmHg	HR b/min	cSBP mmHg	cDBP mmHg	cPP mmHg
Obese	13	4.8	23.52	121.73	73	97.39	88.71	114.33	76.62	39.21
Normal weight	10	4.5	23.9	116.6	73	97.3	82.9	106.2	72.2	35.3
*p*-value		0.95	0.94	0.08	0.19	0.97	0.06	**0.02**	0.5	0.27

The numbers in bold represent statistically significant results.

**Table 12:** PWA parameters comparison in Tanner Stage IV and V subjects.

Tanner IV, V Subjects	*n*	PWV m/s	AIx %	SBP mmHg	DBP mmHg	MAP mmHg	HR b/min	cSBP mmHg	cDBP mmHg	cPP mmHg
Obese	10	4.85	28.33	126.19	82.5	104.3	88.74	118	84.32	36.14
Normal weight	6	4.6	24.6	117	79	96.2	85	103.1	69.1	32.6
*p*-value		0.16	0.5	0.34	0.37	0.17	0.54	**0.02**	**0.002**	0.53

The numbers in bold represent statistically significant results.

**Table 13:** Correlations between waist circumference and PWA parameters in obese and N-weight subjects (Pearson’s r and Spearman’s ρ).

	PWV m/s	AIx %	SBP mmHg	DBP mmHg	MAP mmHg	HR b/min	cSBP mmHg	cDBP mmHg	cPP mmHg
Obese	**ρ = 0.5**	r = 0.24	**ρ = 0.48**	**r = 0.53**	**r = 0.57**	r = −0.07	**ρ = 0.51**	**r = 0.49**	r = 0.11
*p*-value	**0.004**	0.19	**0.007**	**0.003**	**0.001**	0.69	**0.004**	**0.005**	0.54
Normal weight	**r = 0.61**	r = 0.26	**r = 0.62**	ρ = 0.37	**ρ = 0.49**	r = −0.21	**r = 0.48**	r = 0.32	ρ = 0.24
*p*-value	**0.001**	0.19	**0.0009**	0.06	**0.01**	0.32	**0.01**	0.11	0.23

The numbers in bold represent statistically significant results.

**Table 14:** PWA parameters comparison of mean/median values depending on the presence of risk factors in the obese group (*t*-Student tests and Mann–Whitney tests, after checking each parameter’s normality of distribution).

	Risk Factors	*n*	PWV m/s	AIx %	SBP mmHg	DBP mmHg	MAP mmHg	HR b/min	cSBP mmHg	cDBP mmHg	cPP mmHg
Mother’s health during pregnancy	Risk during pregnancy	17	4.8	29.21	121.12	78.53	102.29	90.31	116.04	79.73	38.33
	No risk	13	4.6	19.02	114.07	73.71	95.33	88.11	108.02	76.12	35.52
	*p*-value		**0.02**	**0.009**	**0.01**	0.21	0.053	0.57	0.12	0.32	0.48
Birth weight	<2500 g	7	4.6	22.81	114.04	78.1	99.42	89.82	114.41	78.71	42.08
	>3500 g	8	4.75	28.68	122.11	73.02	98.23	90.31	111.54	75.05	41.55
	Normal	15	4.7	23.63	118.02	77.65	99.62	88.55	113.43	79.54	40.03
	<2500 g vs. N *		0.85	0.88	0.59	0.94	0.96	0.82	0.82	0.85	0.64
*p*-value:	>3500 g vs. N *		0.79	0.26	0.56	0.34	0.94	0.7	0.77	0.33	0.2
	<2500 g vs. >3500 g *		1	0.37	0.59	0.39	0.89	0.92	0.81	0.5	0.37
Postnatal nutrition	Formula	14	4.8	27.11	121.55	78.52	100.03	90.22	112.52	79.84	36.31
	Breastfed	16	4.7	22.82	117.04	74.64	94.73	88.55	113.02	76.66	37.72
	*p*-value		0.39	0.29	0.41	0.32	0.11	0.69	0.44	0.39	0.72
Family history	Risk factors present	20	4.75	27.04	118.52	75.64	99.41	99.14	114.57	76.86	39.52
	No risk factor	10	4.65	20.42	116.05	78.21	99.11	88.45	110.55	80.72	32.21
	*p*-value		0.38	0.96	0.48	0.98	0.92	0.54	0.58	0.33	0.07
Smoking	Smoking	5	5	31.81	125.02	85.82	108.23	93.84	119.04	86.81	32.82
	Passive smoking	10	4.8	26.07	118.07	77.66	99.65	87.22	115.43	78.22	40.44
	No exposure to smoke	20	4.3	22.82	116.04	73.93	97.05	88.71	113.06	75.97	37.34
*p*-value:	Smoking vs. no exposure *		**0.013**	0.12	**0.015**	**0.02**	**0.02**	0.38	0.49	0.03	0.41
	Passive smoking vs. no exposure *		0.06	0.57	0.5	0.48	0.12	0.78	0.64	0.66	0.63
	Smoking vs. passive smoking *		0.67	0.36	0.32	0.18	0.92	0.37	0.66	0.15	0.24
Physical activity	Sedentary	18	4.7	23.55	118.07	77.33	100.55	87.48	113	78.85	38.11
	Normal physical activity	12	4.7	26.84	118.02	75.21	97.51	92.06	112.03	77	35.56
	*p*-value		0.65	0.43	0.6	0.6	0.43	0.28	0.47	0.65	0.52
Sleep	Sleep issues	13	5	24.8	124.98	79.61	103.44	91.13	107.03	76.33	39.72
	Normal sleep	17	4.6	24.82	114.08	74.11	96.25	86.91	117	80.51	35
	*p*-value		**0.004**	0.99	**0.008**	0.16	**0.04**	0.3	**0.017**	0.26	0.22

* These parameters were compared two-by-two, hence the *p*-value for statistical significance is 0.016 (Bonferroni correction). The numbers in bold represent statistically significant results.

**Table 15:** PWA parameters comparison of mean/median values depending on the presence of risk factors in the normal-weight group (*t*-Student tests and Mann–Whitney tests, after checking each parameter’s normality of distribution).

	Risk Factors	*n*	PWV m/s	AIx %	SBP mmHg	DBP mmHg	MAP mmHg	HR b/min	cSBP mmHg	cDBP mmHg	cPP mmHg
Mother’s health during pregnancy	Present	8	4.5	26.12	115.62	79.55	96.52	85.83	102.11	68.58	30
	Not present	17	4.3	19.71	112.63	75.03	94.55	82.77	99.52	68.14	31
	*p*-value		0.29	0.94	0.44	0.11	0.18	0.26	0.57	0.89	0.86
Birth weight	<2500 g *	2	-	-	-	-	-	-	-	-	-
	>3500 g	10	4.7	25.2	111.34	81.5	99.22	83.82	104.87	70.83	31.55
	Normal	13	4.2	19.71	116.77	78	93.51	83.74	98.04	67.11	34.31
	*p*-value		0.048	0.18	0.16	0.18	0.05	0.96	0.12	0.32	0.27
Postnatal food	Formula	9	4.5	24.21	115.35	78	96.33	84.71	103.7	70.21	34
	Breastfed	16	4.4	20.44	112.62	77	94	83.17	98.42	67.14	31.49
	*p*-value		0.6	0.36	0.47	0.73	0.92	0.55	0.22	0.39	0.3
Family history	Risk factors present	7	4.5	25.73	115.56	81	96.51	85.82	102.72	69.35	30
	No risk factor	18	4.4	20.21	112.85	75.5	95	82.84	99.41	67.83	31
	*p*-value		0.39	0.22	0.5	0.08	0.15	0.32	0.49	0.71	0.73
Smoking	Smoking *	2	-	-	-	-	-	-	-	-	-
	Passive smoking	8	4.8	31	118.41	81	99.61	86.41	106.23	73.22	31
	No exposure to smoke	15	4.2	17	110.82	75	92.14	82.11	97	65.53	30.55
	*p*-value		**0.02**	**0.009**	**0.03**	**0.01**	**0.02**	0.12	**0.03**	**0.02**	0.57
Physical activity	Sedentary	8	4.9	28.71	119	81.55	100.21	87.63	106.31	74.84	36.53
	Normal physical activity	17	4.3	18.5	114	75.05	94.55	81.82	97.5	65.14	30
	*p*-value		**0.01**	**0.01**	**0.006**	**0.003**	**0.004**	**0.03**	**0.04**	**0.004**	**0.002**
Sleep	Sleep issues	5	5	27.81	120.81	82.04	100.55	86.22	107.43	72.64	37.62
	Normal sleep	20	4.35	20.33	111.84	76.12	95.72	83.13	98.61	67.25	31.11
	*p*-value		**0.04**	0.13	**0.04**	**0.01**	**0.01**	0.35	0.09	0.2	**0.02**

* For children with a birthweight < 2500 g and for smokers, due to the count of only 2 subjects in the subgroups of study, we did not perform a statistical analysis in which to include them. The numbers in bold represent statistically significant results.

## Abbreviations

Aix	augmentation index
AP	augmentation pressure
BMI	body mass index
BP	blood pressure
cDBP	central diastolic blood pressure
CI	confidence interval
CIMT	carotid intima-media thickness
cPP	central pulse pressure
cSBP	central systolic blood pressure
DBP	diastolic blood pressure
DM	diabetes mellitus
HR	heart rate
MAP	mean arterial pressure
N-weight	normal weight
PWV	pulse wave velocity
PP	pulse pressure
SBP	systolic blood pressure
